# Neuromuscular Compression Garments: Effects on Neuromuscular Strength and Recovery

**DOI:** 10.2478/v10078-011-0055-4

**Published:** 2011-10-04

**Authors:** Martim Bottaro, Saulo Martorelli, José Vilaça

**Affiliations:** 1 Human Performance Laboratory, College of Physical Education, University of Brasília, Brasília, Brazil; 2 Department of Sport Sciences, Exercise and Health, University of Trás-os-Montes and Alto Douro (UTAD), Vila Real, Portugal; 3 Research Center in Sport, Health and Human Development (CIDESD), Vila Real, Portugal

**Keywords:** muscle strength, muscle soreness, recovery, neuromuscular performance

## Abstract

Graduated compression stockings have been used as a mechanical method of deep vein thrombosis prophylaxis for several years. Several studies have demonstrated an increase in mean deep venous velocity, reduced venous pooling, improved venous return, and increase blood lactate clearance in subjects who wore graduated compression stockings during exercise. A possible improvement in venous return during and after exercise may facilitate the clearance of metabolites produced during exercise. Also, studies have suggested that compressive clothing can promote tissue regeneration and consequently positively benefit the muscle function following strenuous exercise. However, the results from the previous studies are controversial. Also, the majority of the studies investigated the effects of compression stockings and there is a lack of studies using different compression garments such as compression shorts, shirts and sleeves. Thus, the purpose of this text is to briefly review the possible effects of compression garments on exercise performance and muscle recovery.

## Introduction

Athletes have been using diverse mechanisms in the attempt of improving their performance during training and competitions. Mechanisms may consist of food supplement ingestion, pharmacological substances, massages among others (Bishop et al., 2008). One mechanism that has been used recently is wearing the proper garment destined to improve the performance during the sport activity (Mollendorf et al., 2004; Kemmler et al., 2009; Tomikawa and Nomura, 2009; Ali et al., 2011). Among the specific clothing, one can observe an exponential increase in the compression garment specially the use of compression stockings (Ali et al., 2007; Sigel et al., 1975; O’Donnell et al., 1979). In the course of time, these stockings would begin to be used with the intention of improving the athletes’ performance (Doan et al., 2003) and accelerate the recovering process (Kraemer et al., 2001; Chatard et al., 2004; Kraemer et al., 2010). Currently, the utilization of different types of compression garment (e.g. pants, shirts, sleeves, etc) has been in evidence in sports that depend more on neuromuscular than cardiovascular performance such as volleyball, tennis, golf, and basketball (Kraemer et al., 1996). Accordingly, in the last decades many studies have been investigating the effects of the use of compression stockings relating to the improvement of the vertical jump (Kraemer et al., 1996), the recovery of the muscular soreness (Kraemer et al., 2001; Kraemer et al., 2010), and the aerobic performance (Chatard et al., 2004; Kemmler et al., 2009; Ali et al., 2011). Therefore, the purpose of this text is to present studies associated to the possible effects of different sportive compression garments in the sports performance.

### Compression Garment: Effects on Cardiovascular and Metabolic Responses

Several studies have investigated the effects of the use of compression garments on cardiovascular and metabolic responses during the aerobic training. Berry and McMurray (1987) evaluated the cardiovascular and metabolic consequences (VO_2max_ and lactate levels) in the use of compression stockings during maximal treadmill tests (n=6) and cycle ergometer (n=6). In the treadmill test, no differences were found in the VO_2max_ or in the blood lactate levels. Yet, in the cycle, the use of compression stockings during and after the test (30 min of recovery) resulted in a smaller concentration of blood lactate when compared to the protocol that used the compression stockings only during the test, and also the same result when compared to the protocol that did not use the stockings at all. In addition, no differences were found between protocols (stocking vs. non-stocking) in the VO_2max_. Almost 10 years later, Bringard et al.(2006) performed a study, divided in two parts. In the first part, they evaluated the energetic cost of the incremental running test in different submaximal intensities (10, 12, 14 and 16 km/h) with six male runners (31.2 ± 5.4 years). The running test was accomplished at 12km/h, in which a lower energetic cost with the use of compression pants was noticed and no difference was perceived in the heart rate, nor in the ventilation and nor in the VO_2max_. In the second part of the study, the slow VO_2_ component of six male individuals was evaluated (26.7 ± 2.9 years) during a 15 min run at 80% of the VO_2max_. The slow component of the VO_2_ was lesser when the subjects used the compression pants (36%). Once again, there was no difference in the heart rate or in the ventilation.

In an investigation of the effects of the compression stockings in 14 male individuals (36.8 ± 11.2 years) with spinal cord injury, Rimaud et al. (2007) performed an incremental test using a wheel chair to measure the lactate levels, the heart rate, the blood pressure, the VO_2_ and the maximum capacity during the test. A statistical difference was found only in the blood lactate levels, which was lesser in the compression stockings’ test. Ali et al. (2007) conduced a study divided in two parts. In the first part, 14 male individuals (22 ± 0.4 years) realized 20 meters consecutive running tests with progressive velocity, and in the second part a 10 km run was performed. No differences were found in the heart rate or the rate of perception exertion (RPE) in none of the studies. Only in the second study the delay of muscle soreness (DOMS) was less intense 24 hours later, when the volunteers used graduated compression stockings.

In 2010, Sear et al. investigated the use of full body compression garment during continuous low intensity running and intermittent activity of high intensity. Eight athletes (20.6 ± 1.2 years) participated of the test. The use of this specific garment improved the performance during the low intensity run resulting in a bigger distance covered at the end of the test (4.21 ± 0.51 km vs. 4.56 ± 0.57 km). Also, there was an improvement of the muscular oxygenation (53.5 ± 8.3% against 55.8 ± 7.2%). No differences were found in the blood lactate concentrations and in the VO_2max_. Hence, it follows that there are still some controversies about the effects of the compression garment utilization in order to improve the aerobic performance and the removal of blood lactate.

### Compression Garment: Effects on Neuromuscular Response

One of the first studies to verify the neuromuscular responses resulting from the use of compression shorts was Kraemer et al. (1996). Here, 18 male individuals (21.2 ± 3.1 years) and 18 female individuals (20.4 ± .09 years) all volleyball college players were evaluated. In this study, a power test of 10 consecutive jumps was performed, with a three-second interval between the jumps. No differences were found in the maximum power produced during the jumps. However, there was an increase of the average power when the volunteers used the compression shorts. Doan et al. (2003) evaluated diverse parameters of performance of 10 male individuals (20.0 ±0.9 years) and 10 female individuals (19.2 ± 1.3 years) in tests using the compression and tests without the compression garment (60 meters sprint and vertical jump). No time differences were found during the running test. Yet, subjects that used the compression garment jumped high (0.461 vs. 0.485 m) and showed statistical difference during the protocols.

More recently, Duffield and Portus (2007) evaluated the utilization of three types of full body compression garment in the distance of consecutive throws of ten cricket players (22.1 ± 1.1 years) before and after 20 meters running tests (intervals of 30 min). No differences were found in the performance during the run or in the total distance of the throws during the tests. All the compression garments were capable of reducing the delayed onset of muscle soreness (DOMS) in the upper and lower limbs 24 hours after the tests. Soon after that, Duffield et al. (2008) evaluated the use of compression shorts during and after two consecutives test sessions (with a 24h interval) which simulated a rugby match with 14 young rugby athletes. The time of the 20 meters running test was evaluated concurrently with the maximum power test, the DOMS and the concentration of creatinine kinase in the blood. In this case, no difference was found relating to time, but the athletes reported a lesser muscle pain when using the compression shorts during the tests and in the recovery period. At the same time, Scanlan et al. (2008) performed a incremental test and a one hour trial duration one time with and one without the compression pants. The experiment was constituted of 12 male cyclists (20.5 ± 3.6 years). No statistical differences were found in the power or in the muscular oxygenation during the incremental test or also during the one-hour timing trial.

In 2010, Duffield et al. evaluated 11 athletes (2.9 ± 2.7 years) who performed 20 meters runs and horizontal jumps during 10 min, again evaluated with and without the compression pants, during the experiment and 24 hours after the test. No differences in the timing were noticed, nor in the distance of the horizontal jumps, nor in the power of the extensors and flexors of the knee, or in the RPE, nor in the creatine kinase concentration, and nor in the reactive protein-C and blood pH. Only the DOMS showed some reduction 24 hours after the execution of the tests with the compression pants. In the same year, Ali et al. (2010) performed an experiment with nine male and one female individual (36 ± 10 years) to evaluate the effects of the graded compression stockings (high and low compression) during a 10 km run, in jumps executed before and after the run, and in the concentration of blood creatine kinase and lactate. No significant differences were found in the performance during the run, nor in the high and power of the jumps prior to the test and after, and neither in the concentration of the blood markers.

Kraemer et al. (2010) evaluated the use of full body compression garment for a 24h period, after a high intensity session of high resistance training with 11 male individuals (23 ± 2.9 years) e nine female individuals (23.1 ± 2.2 years). The results demonstrated that the recovery achieved with the utilization of compression garment resulted in less fatigue, muscular pain and muscular swelling, and a better performance in the bench press power test (throwing). No statistical difference was found for the other analyzed variables (Sleep quality, reaction time and blood creatine kinase). In a more recent study, Ali et al. (2011) used nine male and three female trained individuals to evaluate the effects of the graded compression stockings, tested with different levels of compression (low, medium and high compression), during a 10 km run and in jumps performed prior and after the run. No difference was found during the running performance. However, medium and low compression stockings showed a smaller variation in the high of the jumps prior and after the run.

As one can see, there is still much controversy among studies about the ergogenic effects in the utilization of compression garment in the aerobic performance and in the neuromuscular responses. It appears that the compression garment can be more effective in the recovery of the muscular damage after the exercise. The removal of blood lactate also appears to be favored by some kind of compression clothing. Therefore, coaches and athletes should take advantage of these resources in order to help their athletes, especially during championships games executed with small rest between them, which do not allow full recovery. Also, although many sports are using compression sleeves (i.e. basketball, golf, tennis, stand up paddle), the majority of the studies in the literature focus on investigate only the use of compression stockings. In this sense, new studies need to be realized with other types of compression garments such as pants, shorts and specially sleeves to better establish their possible effects.
